# The Vasopressin Receptor Antagonist Tolvaptan Counteracts Tumor Growth in a Murine Xenograft Model of Small Cell Lung Cancer

**DOI:** 10.3390/ijms25158402

**Published:** 2024-08-01

**Authors:** Laura Naldi, Benedetta Fibbi, Simone Polvani, Chiara Cirillo, Francesca Pasella, Francesca Bartolini, Francesca Romano, Alessandra Fanelli, Alessandro Peri, Giada Marroncini

**Affiliations:** 1 Pituitary Diseases and Sodium Alterations Unit, AOU Careggi, 50139 Florence, Italy; laura.naldi@unifi.it (L.N.); alessandro.peri@unifi.it (A.P.); giada.marroncini@unifi.it (G.M.); 2 Endocrinology, Department of Experimental and Clinical Biomedical Sciences “Mario Serio”, University of Florence, AOU Careggi, 50139 Florence, Italy; chiara.cirillo1@edu.unifi.it (C.C.); francesca.pasella@edu.unifi.it (F.P.); francesca.bartolini9@edu.unifi.it (F.B.); 3 Gastroenterology Unit, Department of Experimental and Clinical Biomedical Sciences “Mario Serio”, University of Florence, 50139 Florence, Italy; simone.polvani@unifi.it; 4 Central Laboratory, Careggi University Hospital, 50139 Florence, Italy; romanof@aou-careggi.toscana.it (F.R.); fanellia@aou-careggi.toscana.it (A.F.)

**Keywords:** tolvaptan, vasopressin receptor, small cell lung cancer, hyponatremia

## Abstract

We have previously demonstrated that the vasopressin type 2 receptor (AVPR2) antagonist tolvaptan reduces cell proliferation and invasion and triggers apoptosis in different human cancer cell lines. To study this effect in vivo, a xenograft model of small cell lung cancer was developed in Fox1^nu/nu^ nude mice through the subcutaneous inoculation of H69 cells, which express AVPR2. One group of mice (n = 5) was treated with tolvaptan for 60 days, whereas one group (n = 5) served as the control. A reduced growth was observed in the tolvaptan group in which the mean tumor volume was significantly smaller on day 60 compared to the control group. In the latter group, a significantly lower survival was observed. The analysis of excised tumors revealed that tolvaptan effectively inhibited the cAMP/PKA and PI3K/AKT signaling pathways. The expression of the proliferative marker proliferating cell nuclear antigen (PCNA) was significantly lower in tumors excised from tolvaptan-treated mice, whereas the expression levels of the apoptotic marker caspase-3 were higher than those in control animals. Furthermore, tumor vascularization was significantly lower in the tolvaptan group. Overall, these findings suggest that tolvaptan counteracts tumor progression in vivo and, if confirmed, might indicate a possible role of this molecule as an adjuvant in anticancer strategies.

## 1. Introduction

Hyponatremia is present in up to 40% of hospitalized cancer patients and is the most frequent electrolyte disorder in this condition [1,2]. Several factors may determine a reduced serum sodium concentration ([Na^+^]) in cancer, including vomiting, diarrhea, and intravenous hydration during chemotherapy, or comorbidities, such as heart failure, renal failure, and liver cirrhosis [3,4,5,6]. However, the most frequent etiology of hyponatremia in cancer patients is the Syndrome of Inappropriate Antidiuresis (SIAD) [7]. SIAD may be due to the tumoral secretion of arginine vasopressin (AVP), which occurs most frequently in lung cancer [3], or may be induced by drugs (e.g., chemotherapeutics, opioids), nausea, or pain [4,8,9,10].

There is evidence that hyponatremia, independently of etiology, negatively affects the outcome of cancer patients [8,11]. This observation has been supported by experimental data, which demonstrated that in cancer cells from different tumors (i.e., small cell lung cancer (SCLC), pancreatic adenocarcinoma, neuroblastoma, and colorectal adenocarcinoma, chronic myeloid leukemia), cultured in low [Na^+^], both the proliferation rate and the invasion potential were markedly increased, compared to cells maintained in normal [Na^+^] [11,12]. These results were further supported by the demonstration that hyponatremia secondary to SIAD significantly increased cancer growth in a murine xenograft model of neuroblastoma by activating molecular mechanisms that promote proliferation, angiogenesis, and invasiveness [13].

Among the strategies used for the treatment of hyponatremia, a distinguished role has been recognized in the last fifteen years in AVP receptor type 2 (AVPR2) antagonists, the so-called vaptans. This new class of drugs has been approved in the U.S. and in Europe for the treatment of SIAD-related hyponatremia. Tolvaptan, the most widely used vaptan, effectively induces water diuresis and corrects hyponatremia in SIAD secondary to different etiologies, including cancer [14,15,16,17,18,19]. It is noteworthy that the correction of hyponatremia has been associated with a better outcome in cancer patients [20,21,22].

Interestingly, tolvaptan was also shown to inhibit the production of renal cell cAMP and to counteract disease progression in an animal model of autosomal dominant polycystic kidney disease (ADPKD) [23]. Based on this finding, clinical trials on the possible beneficial effect of tolvaptan for the treatment of ADPKD were performed and showed that this drug effectively reduces renal cyst growth and the progression to end-stage renal failure [24,25]. These studies led to the approval of tolvaptan for a different indication, in addition to SIAD.

We and other groups have demonstrated that tolvaptan markedly reduces cell proliferation and invasiveness, whereas it triggers apoptosis in different tumors, such as SCLC, neuroblastoma, colorectal adenocarcinoma, hepatocarcinoma, and clear cell renal cell carcinoma (ccRCC) [12,26,27,28].

The aim of the present study was to determine the in vivo effects of tolvaptan on the growth of SCLC H69 cells implanted in Foxn1^nu/nu^ mice.

## 2. Results

### 2.1. Weight and Serum Parameters of Mice

**Figure 1:**
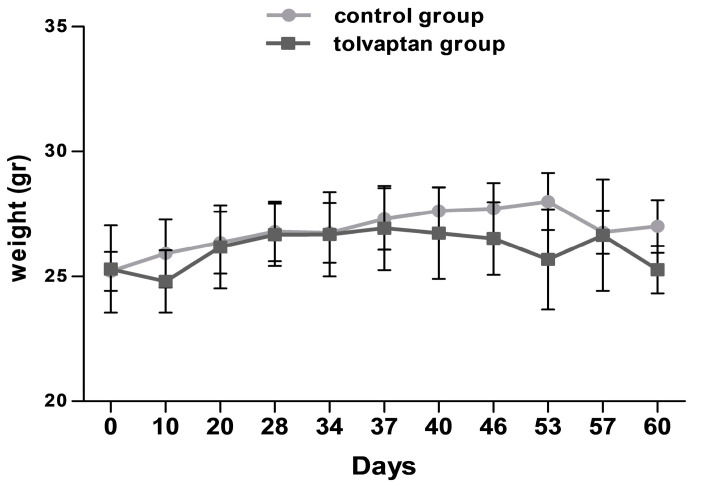
Body weight measurements at different time points in control group and tolvaptan group.

Weight and serum parameters were analyzed at sacrifice. No difference in mice weight was observed between the control (27.0 ± 1.8 gr) and the tolvaptan group (25.6 ± 1.6 gr) (Figure 1). Serum [Na^+^] was unchanged (152.3 ± 0.5 mEq/L in the control group and 154.1 ± 4.1 mEq/L in the tolvaptan group). These values are to be considered normal [Na^+^] in this mouse strain. Hepatic (ALT and AST) and renal (creatinine, blood nitrogen urea) parameters were unaltered (Table 1).

### 2.2. Tumor Growth

**Figure 2:**
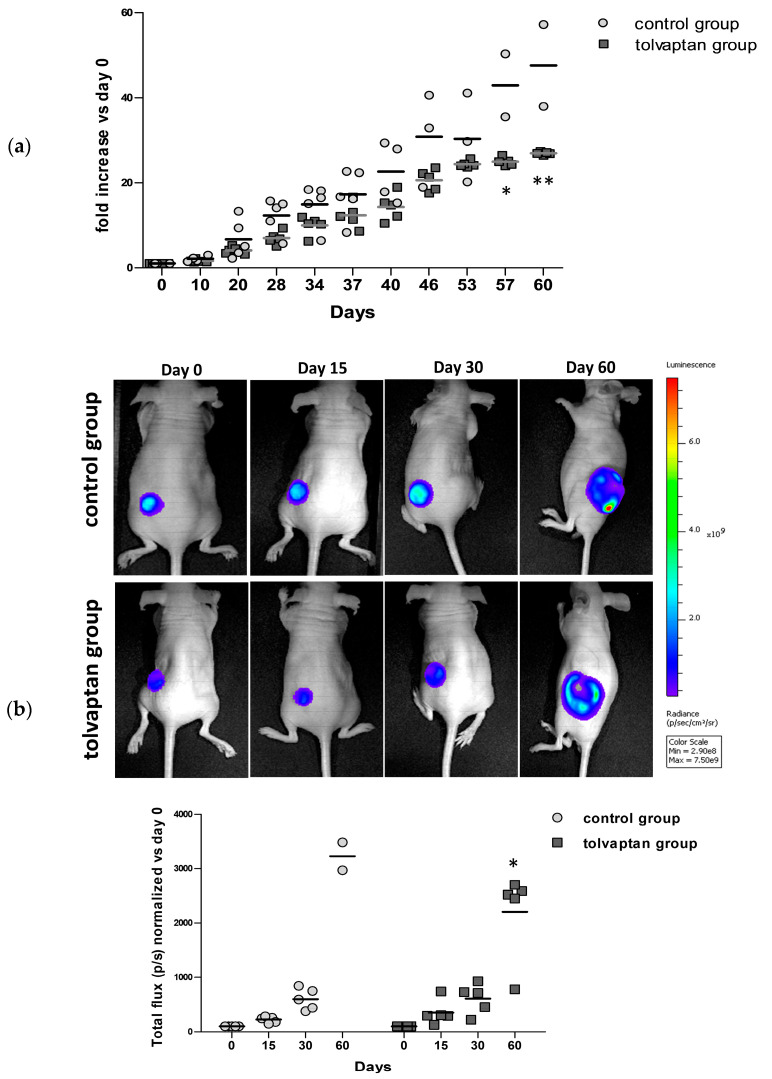
Tumor growth. (**a**) Tumor masses were measured at different time points (* = *p* ≤ 0.05, ** = *p* ≤ 0.02 vs. control group, n = 5 for each group) expressed as fold increase compared to day 0. (**b**) Representative images of bioluminescence imaging after intraperitoneal injection of Luciferin (100 μL/10 gr) in one control and one tolvaptan-treated mouse at different time points (day 0, 15, 30, and 60). Images were obtained with identical experimental conditions (luminescence scale on right). Scatter plot represents total flux (p/s) of bioluminescence emissions of normalized tumor masses vs. day 0 (* = *p* ≤ 0.05 vs. control group, n = 5 per group).

Fox1^nu/nu^ mice were subcutaneously implanted with H69 Luc2-positive cells and tumor growth was monitored for 60 days. Two different operators independently measured tumor masses using a digital caliper at different time points and the progression of growth for each time point is represented in Figure 2. Notably, after the first 10 days, tumors from tolvaptan-treated mice started showing a reduced tumor growth compared to controls; the difference between the two groups, expressed as the fold increase (compared to the initial volume of 100 mm^3^), became statistically significant on day 57 (39.9 ± 5.2 in the control group and 25.2 ± 0.5 in the tolvaptan group) and 60 (46.6 ± 6.0 in the control group and 26.9 ± 0.2 in the tolvaptan group) (*p* ≤ 0.05 and *p* ≤ 0.02, respectively) (Figure 2a). Similarly, tumor activity, assessed in vivo with the IVIS Lumina S5 System, was considerably different between the two groups, and bioluminescence emission became significantly different on day 60 (*p* ≤ 0.05 vs. control group) (Figure 2b).

### 2.3. Mice Survival and Analysis of Excised Tumor Masses

**Figure 3:**
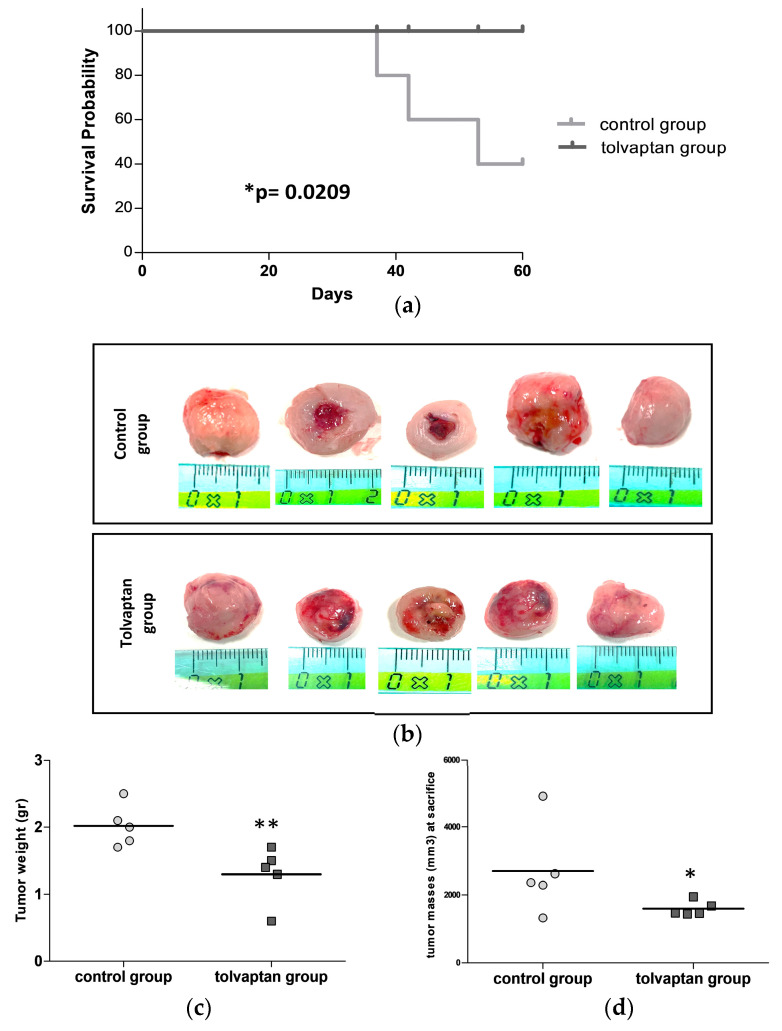
Survival analysis and tumor mass measurement. (**a**) Kaplan–Meier curves show the percent survival of control and tolvaptan groups. (**b**) Images of tumor masses excised at sacrifice from each animal of the two groups. (**c**) Scatter plot of tumors’ weight at sacrifice. (**d**) Scatter dots plots of tumors’ volume at sacrifice. (* = *p* ≤ 0.05 and ** = *p* ≤ 0.02 vs. control group, n = 5 per group).

The Kaplan–Meier curves showed a statistically significant difference in overall survival between the control group (47.6 ± 12.9 days) and the tolvaptan group (60.0 ± 0 days) (*p* ≤ 0.02, log-rank test). Indeed, three of five animals in the control group had to be sacrificed prematurely due to the ulceration of the masses, a condition that was one of the HEP to be fulfilled (Figure 3a). At sacrifice, tumor masses were extracted, and their weight and volume were precisely measured. Mice belonging to the tolvaptan group displayed a markedly lower tumor weight and volume in comparison to the control group (*p* ≤ 0.02 vs. control group) (Figure 3b,c). Notably, the tumor volume at sacrifice reached 2704.6 ± 593.5 mm^3^ in the control group and 1604.8 ± 95.6 mm^3^ in the tolvaptan group (Figure 3d).

### 2.4. Cell Signaling Pathways Affected by Tolvaptan

**Figure 4:**
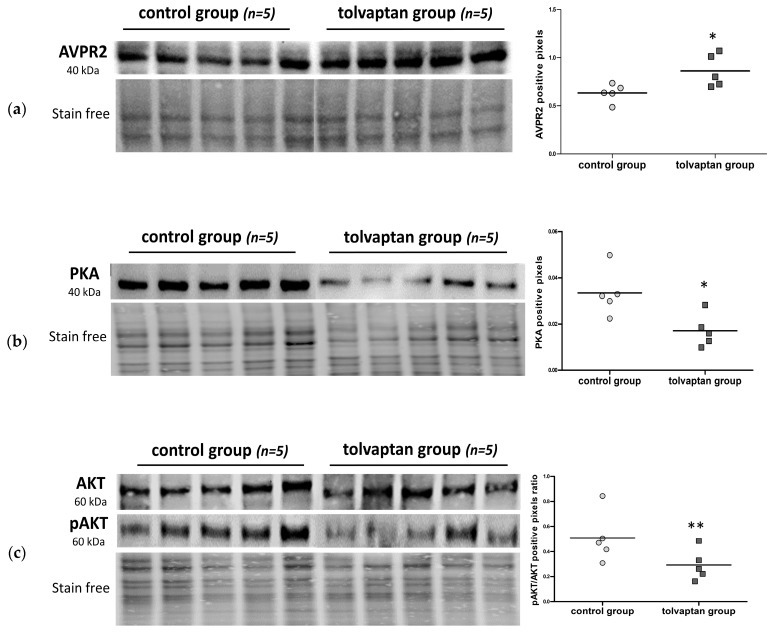
Analysis of cell signaling pathways. (**a**) AVPR2, (**b**) PKA, and (**c**) AKT and pAKT protein levels in the control and treated tumors (n = 5 per group), as assessed by Western blot analysis. Dot blots on the right show their quantification. The results in the graphs represent the protein expression of AVPR2 and PKA and the normalized pAKT/AKT ratio vs. stain-free blot gels, expressed in arbitrary units (* = *p* ≤ 0.05 and ** = *p* ≤ 0.02 vs. control group).

It was previously demonstrated that H69 cells express AVPR2 [12]. Here, we confirmed the expression of the transmembrane receptor in the excised tumors. AVPR2 expression was greater in the tolvaptan group (*p* ≤ 0.05 vs. control group) (Figure 4a). The interaction between tolvaptan and AVPR2 is expected to inhibit the cAMP-PKA pathway. Actually, tolvaptan treatment induced a statistically significant reduction in PKA expression (*p* ≤ 0.05 vs. control group) (Figure 4b). The PI3K/AKT proliferative pathway was also analyzed. Although total AKT remained unchanged between the two groups, its Ser473-phosphorylated (i.e., activated) form was significantly lower upon tolvaptan treatment (*p* ≤ 0.02 vs. control group) (Figure 4c).

### 2.5. Proliferative Potential and Apoptosis Analysis

**Figure 5:**
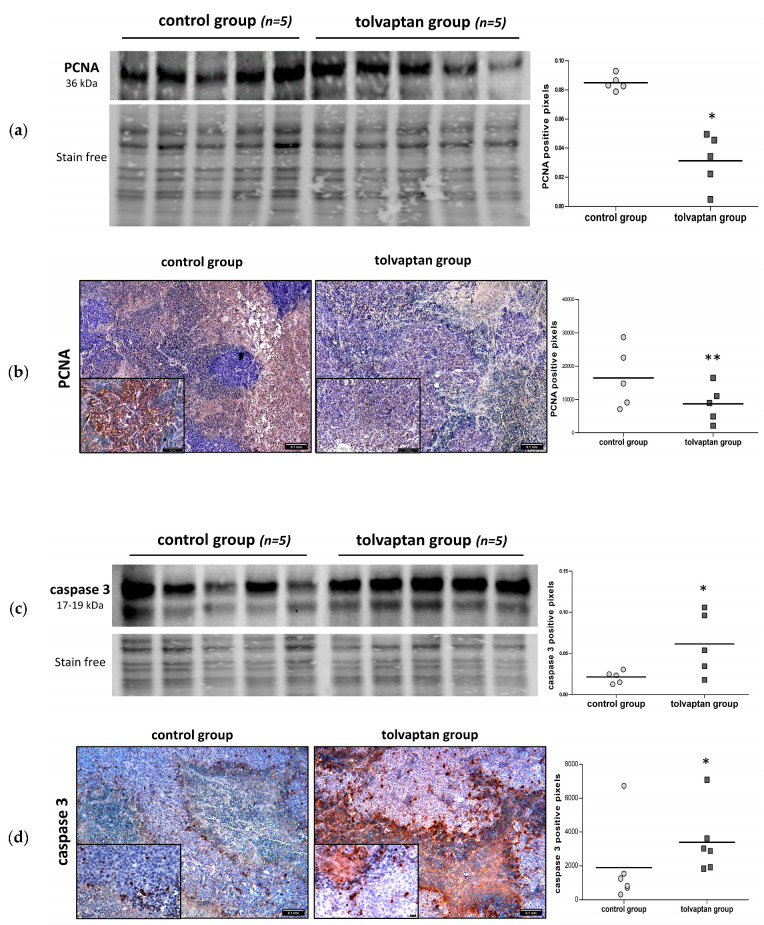
Western blot and IHC analysis for PCNA and caspase-3. (**a**) Western blot analysis of PCNA protein expression in tumor masses of control and tolvaptan mice: images represent blots, whereas scatter dot plots on right show normalized PCNA vs. stain-free gel (* = *p* ≤ 0.05 vs. control group). (**b**) PCNA IHC on tissue sections obtained from tumors: representative images are shown on left, whereas scatter plot on right illustrates the densitometric analysis of PCNA-positive cells (** = *p* ≤ 0.02 vs. control group). (**c**) Western blot analysis of caspase-3 in tumor masses of control and tolvaptan mice: images on left represent blots, whereas scatter dot plots on right show the densitometric analysis of normalized protein expression vs. stain-free gel (* = *p* ≤ 0.05 vs. control group). (**d**) Caspase-3 IHC of tissue sections obtained from tumors: representative images are shown on left, whereas scatter dot blots on right illustrate densitometric analysis of caspase-3-positive cells (* = *p* ≤ 0.05 vs. control group).

The proliferative potential of tumor masses was assessed by analyzing the expression of PCNA through Western blot and IHC. In tumors excised from tolvaptan-treated animals, the PCNA expression was significantly lower than that in tumors from control mice (*p* ≤ 0.05 vs. control group) (Figure 5a,b). The expression of caspase-3, a key apoptotic marker, was also assessed. The total caspase-3 protein expression, as assessed by Western blot, was greater in tolvaptan-treated mice compared to control animals (Figure 5c). Similar results were observed by IHC analysis (*p* ≤ 0.05 vs. control group) (Figure 5d).

### 2.6. Tumor Mass Fibrosis and Angiogenesis

**Figure 6:**
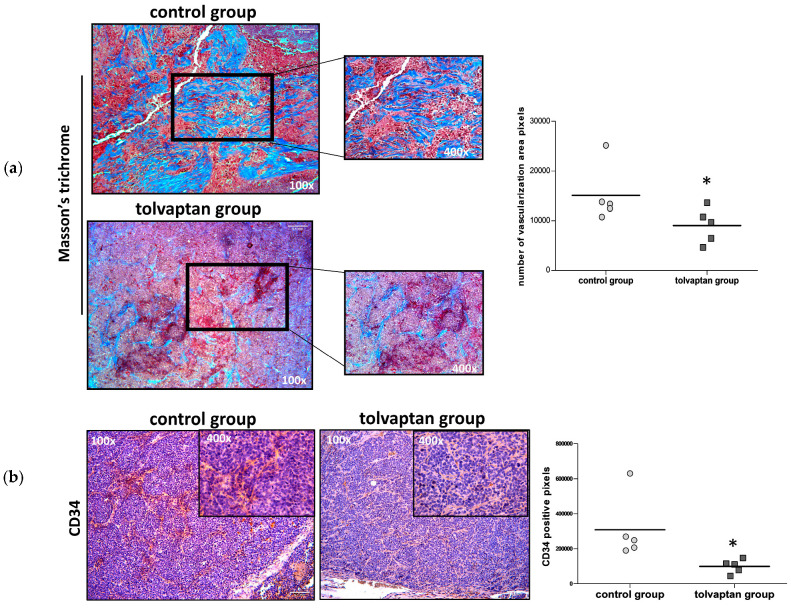

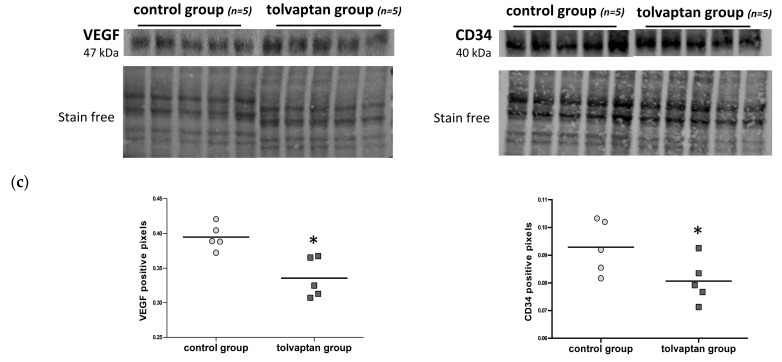
Masson’s trichrome staining and angiogenesis evaluation. (**a**) Masson’s trichrome staining labels collagen in blue, nuclei in dark brown, muscle tissue in red, and cytoplasm in pink. Images are representative of tumor masses of control and tolvaptan groups, whereas dot blots represent mean of tumoral fibrotic area (number of blue pixels) from each group (magnification 100× and 400×) (n = 5). (**b**) IHC analysis of CD34: images are representative of positive stain and the graph shows relative quantification (magnification 100× and 400×) (n = 5). (**c**) Western blot analysis of VEGF and CD34 and their normalized quantification vs. stain-free (n = 5). (* = *p* ≤ 0.05 vs. control group).

Masson’s trichrome staining was used in tissue sections in order to analyze collagen fibers and to determine whether there was any evidence of vascular remodeling. Using this approach, the proportion of stroma appeared more represented in the control group than in the tolvaptan group (*p* ≤ 0.05) (Figure 6a). Moreover, the vascularized area appeared less evident in tolvaptan-treated mice as confirmed by anti-CD34 staining (*p* ≤ 0.05 vs. control group) (Figure 6b). Reduced vascularization and angiogenesis were further confirmed by Western blot analysis for CD34 and VEGF. Indeed, significantly lower expression levels of both biomarkers were observed in tumor masses from tolvaptan-treated mice compared to control mice (*p*≤ 0.05) (Figure 6c).

## 3. Discussion

The unpredicted anti-proliferative effects of vaptans, initially licensed for the treatment of hyponatremia secondary to SIAD, opened a new scenario on a possible role of this class of drugs in counteracting tumor growth [12,26]. In this study, we evaluated the inhibitory effect of tolvaptan on the growth of an SCLC cell line in a murine xenograft model. The choice of SCLC was based on the fact that, although tumors from different organs and tissues may ectopically secrete AVP and cause SIAD [8], SCLC is the tumor where the probability that patients experience at least one episode of hyponatremia is highest (>75%) [29]. Nevertheless, overall, the possibility that hyponatremia in a cancer patient is secondary to SIAD due to ectopic AVP secretion is >30% [30]. Besides the symptoms that can be elicited by hyponatremia, it is well established that this electrolyte imbalance is a negative prognostic factor in cancer patients [11]. Therefore, targeting AVPR2 might have a beneficial effect both on hyponatremia, when present, and on tumor growth.

Tolvaptan treatment did not modify body weight nor induce dehydration, as confirmed by the urea/creatinine ratio, which did not differ between the tolvaptan-treated and control groups. These findings indicate that the aquaretic effect of tolvaptan was effectively counteracted by water consumption in treated mice. Furthermore, serum [Na^+^] remained in the physiological range for the mouse strain Fox1^nu/nu^ and did not differ between the two groups, thus excluding the possibility that differences in tumor growth might be influenced by hyponatremia in the control group. A possible hepatotoxic effect has been described in patients treated with tolvaptan for ADPKD, in which higher doses than those used for hyponatremia are administered [31]. However, in our study, serum levels of transaminases remained in the normal range and did not differ between the two groups of animals.

It is noteworthy that in tolvaptan-treated animals, tumor growth was significantly inhibited compared to the control ones. Specifically, at the experimental endpoint, a significant difference in the fold increase in tumor masses, which was almost two-fold lower in the tolvaptan group vs. the control group, was observed. The fact that cells had been transfected with luciferase allowed the detection of bioluminescence emission by tumoral masses. The bioluminescence emitted by tumors in mice treated with tolvaptan was significantly lower, thus confirming the effectiveness of this molecule in arresting tumor growth. Accordingly, the tumor volume and weight at the time of sacrifice were significantly lower in the tolvaptan group.

The effect of tolvaptan in counteracting tumor growth was also confirmed by the significantly reduced survival observed in the control group. In particular, all tolvaptan-treated mice were alive at the end of the experimental timeframe, whereas three out of five mice in the control group had to be sacrificed earlier, due to the presence of ulcerations, according to the established HEP.

At sacrifice, tumors were excised to perform further analyses on tissue samples. We had previously shown that the H69 cell line express the AVPR2 [12]. Here, we confirmed the expression of the transmembrane receptor in the tumors excised from both groups of animals. In addition, the amount of the AVPR2 protein in the tolvaptan group was significantly higher. Admittedly, the reason behind such a difference remains unclear. However, the activation of the AVPR2 by AVP, which is secreted by H69 cells [32], has been associated with an increased cell proliferation rate in ccRCC, both in in vitro as well as in in vivo experiments [27,33]. Thus, it is tempting to speculate that the increased expression of AVPR2 in tumors of tolvaptan-treated mice might be viewed as an attempt to counteract the anti-proliferative effect of tolvaptan.

In agreement with the intracellular signaling pathway activated by AVP through the AVPR2, tolvaptan determined a reduction in the expression of PKA and in the pAKT/AKT ratio. These data are consistent with those obtained in our previous in vitro studies [11,12].

Further analyses were performed in excised tumor tissues in order to assess proliferative and apoptotic markers. Specifically, the expression of the proliferative marker PCNA was analyzed in tumors from the two groups of animals. PCNA is a nuclear protein and is involved in DNA replication, elongation, and repair [34]. It also has a role in cell cycle progression by interacting with cyclin/cdk, and it has been proposed as a molecular target for antitumoral treatments [35,36]. Significantly lower levels of PCNA were found in tumors from animals treated with tolvaptan compared to tumors from the control group. Conversely, the expression of caspase-3, an important mediator of apoptosis [37], was markedly higher in tumors from the tolvaptan group. Accordingly, tolvaptan significantly reduced vascularization in tumor masses, as supported by the lower expression levels of VEGF and CD34 in tumors excised from tolvaptan-treated animals. These findings reinforce the hypothesis of an antitumoral effect of the drug, because vascularization plays an essential role in promoting tumor growth. Specifically, VEGF is a well-known growth factor that stimulates vascular endothelial cell proliferation in physiologic as well as in pathologic conditions [38]. Consequently, VEGF receptors have been identified as potential targets in anticancer strategies, and an increasing number of VEGF receptor inhibitors have been discovered and used in clinical practice [39]. On the other hand, the CD34 protein was identified about forty years ago as a marker of hematopoietic stem cells [40] and is currently used as a tool to assess angiogenesis in malignancies [41].

Overall, these findings provide a molecular explanation for the macroscopic observation that tolvaptan effectively reduced tumor growth in our murine xenograft model. Admittedly, our study included a limited number of animals. However, another study has shown that tolvaptan, at a virtually identical dose to that used in our study, inhibited cell proliferation and angiogenesis, yet increased apoptosis, in a mouse xenograft model of renal cancer, whereas desmopressin (dDAVP), an AVP analog, significantly increased cell proliferation [27]. Similarly, an additional study demonstrated opposite effects of dDAVP and satavaptan, another AVPR2 antagonist, in renal cancer cell lines [33]. Furthermore, the same dose of tolvaptan used in our study was also shown to exert the maximal effect against the onset of end-stage renal failure in an animal model of ADPKD [42]. It has to be said that this issue remains to be completely elucidated, because other studies suggested that desmopressin has an anti-proliferative effect in cancer cells [43,44,45]. Admittedly, there are many factors that may influence the cell response to specific molecules. In particular, in the case of AVPR2 in cancer cells, it can be hypothesized that cell behavior may differ, depending for instance on the type of cancer or on the possible presence of receptor mutations [43,46,47,48].

Interestingly, as mentioned previously, tumors may produce and secrete AVP, in addition to expressing AVPR2, thus favoring self-maintenance and proliferation of the tumor itself. The SCLC H69 cell line represents an example of such a situation, because these cells express the AVPR2 and also secrete AVP [46]. In such a scenario, tolvaptan may be viewed as a strategy aiming to break this autocrine loop. In view of increasing evidence supporting this hypothesis [12,13], which appears reinforced by our study, it is reasonable to suggest that AVPR2 antagonism might have a dual role in anticancer strategies, through the correction of hyponatremia, when present, and through a direct anti-proliferative effect. Nevertheless, additional pre-clinical studies are needed in order to confirm these preliminary data and to further clarify the molecular mechanisms that are involved in AVPR2 antagonism, as well as the lowest effective dose of tolvaptan against cancer progression.

## 4. Materials and Methods

### 4.1. Chemicals and Reagents

Human small cell lung cancer cell line (NCI-H69, 91091802), RPMI-1640 culture medium, fetal bovine serum (FBS), L-glutamine, antibiotics (penicillin and streptomycin), and Hank’s Balanced Salt Solution (HBSS) were purchased from Millipore (Milan, Italy). Tolvaptan was provided by Otsuka Pharmaceutical (Samsca, Otsuka Pharmaceutical Italy, Milan, Italy).

### 4.2. Cell Culture and Transfection of H69 Cells

H69 cells were cultured in completed RPMI-1640 culture medium and maintained at 37 °C in a humidified atmosphere (5% CO_2_/95% air). Luciferase-expressing H69 cells were produced following the Effectene^®^ Transfection Reagent kit (301425, QIAGEN, Hilden, Germany) protocol. Briefly, 2 × 10^6^ cells were cultured in six-well plates. When 80% confluence was reached, H69 cells were transfected with the pGL4.51 plasmid (Luc2/CMV/Neo) (Promega Corporation, Madison, WI, USA) using the Enhancer (301425, QIAGEN, Hilden, Germany) and the Effectene^®^ Transfection Reagent (301425, QIAGEN, Hilden, Germany) as per protocol. Seventy-two hours after transfection, the cells were placed in complete medium supplemented with geneticin at the optimal concentration of 600 µg/mL (G418, 108321-42-2, Invivogen, San Diego, CA, USA).

### 4.3. Setup of a Xenograft Mouse Model of SCLC

All animal experiments were conducted in accordance with institutional ethical standards and national laws after approval by the Ministry of Health [D. No. 512/2022-PR (prot. 17E9C.261)]. This study involved eight-week-old female Foxn1^nu/nu^ mice (n = 10) obtained from Charles River Laboratories International (Wilmington, MA, USA). Mice were housed in a standard animal facility (Ce.S.A.L, Neurofarba, University of Florence, Italy) in sterile areas with ventilation and sterile barriers. The facility was equipped with a 12/12 h light/dark cycle and a constant temperature (21–23 °C), and the mice were housed in “sterile filter top” cages.

After the first period of acclimatization, mice were subcutaneously implanted with 2 × 10^6^ H69 Luc2-H69 cells on one flank. The tumor volume (mm^3^) was monitored daily using a digital caliper (0.52 × long side × (short side)^2^). Upon reaching a tumor volume of approximately 100 mm^3^, mice were caged individually and randomly assigned to the control group (n = 5) or the tolvaptan group (n = 5). To facilitate drug administration, each mouse was fed 20–25 g/day of liquid diet at 1.0 kcal/mL of caloric intake (Rodent Liquid Diet AIN-76A, Mucedola S.R.L., Milan, Italy), consisting of 66% carbohydrate, 21% protein, 12% fat and vitamins, and water ad libitum for the entire experimental period (60 days). The tolvaptan group received tolvaptan added to the daily liquid diet at a concentration of 130 mg/kg. All the daily food was ingested. Mice were sacrificed on day 60 or when they reached the Humane Endpoint (HEP) criteria (e.g., cachexia, loss of weight ≥ 20%, epilepsy, inability to move, tumor ulceration) following the Italian Health Ministry protocol.

### 4.4. In Vivo Imaging

In addition to the digital caliper, tumor growth was also assessed using the Lumina IVIS S5 imaging system (Perkin Elmer, Waltham, MA, USA) on the first day of the experiment (day 0), on day 15, on day 30, and on the day of sacrifice. Before imaging, mice received an intraperitoneal injection of D-luciferin potassium salt solution (100 µL/10 g of body weight at a concentration of 15 mg/mL, Perkin Elmer, Waltham, MA, USA). Subsequently, after anesthesia with 2.5% isoflurane (1 L/min flow), bioluminescent images were acquired using the Lumina IVIS S5 (Department of Experimental and Clinical Biological Sciences “Mario Serio”, Florence, Italy). Luminescence was measured as radiance (total flux photon/sec) with the Living Image^®^ 4.7.2 Software (Perkin Elmer, Waltham, MA, USA) in the region of interest (ROI) encompassing tumor masses.

### 4.5. Serum Analysis

At sacrifice, mice were euthanized using an overdose of anesthesia (ketamine/xylazine) to perform transthoracic beating heart cardiocentesis. The collected blood samples were then centrifuged at 3000× *g* for 10 min at 4 °C and processed for serum [Na^+^], hepatic parameter [(alanine transaminase (ALT), aspartate transaminase (AST)], and renal parameter (urea and creatinine) analyses using the Cobas 8000 analyzer (Roche/Hitachi family, Basel, Switzerland). Serum analyses were carried out by the General Clinical Chemical laboratory of Careggi University Hospital (Florence, Italy) according to standard procedures.

### 4.6. Tissue Preparation and Masson’s Trichrome Analysis

Upon sacrifice, tumor masses were promptly explanted from the primary cancer site, measured, weighed, and fixed in 10% formalin (65-30001F, Bio-Optica Milano Spa, Milan, Italy). Subsequently, they were embedded in paraffin using a tissue embedding system (ASP300S and HistoCore processor, Arcadia Inclusion System, Leica Biosystems, Milan, Italy). Tumor mass sections (5 µm thick) were stained with Masson’s trichrome (MT) staining procedure (14-118 DDK Italia S.r.l., Milan, Italy) according to the manufacturer’s instructions. Aniline blue-stained areas, indicative of collagen fibers, were analyzed and quantified using ImageJ (https://fiji.sc, accessed on 1 April 2024) and GraphPad Prism 5.0 Software (https://www.graphpad.com, accessed on 1 April 2024).

### 4.7. Immunohistochemical (IHC) Analysis of Tumor Masses

The de-paraffinized and rehydrated sections were boiled in citrate buffer (pH = 6) at 95 °C for 10 min to unmask the antigen, treated with 6% H_2_O_2_ solution to inhibit tissue endogenous peroxidases, and blocked in PBS/BSA 2% solution for 1 h. In addition, the ReadyProbes™ Mouse-on-Mouse IgG Blocking Solution (R37621, Invitrogen, Waltham, MA, USA) was used to block endogenous mouse antibodies. The incubation with primary antibodies was carried out overnight at 4 °C. The primary antibodies used were mouse monoclonal anti-Proliferating Cell Nuclear Antigen (PCNA) (#2586S, 1:100, Cell Signaling Technology, Danvers, MA, USA), rabbit polyclonal anti-caspase-3 (#9661, 1:100, Cell Signaling Technology, Danvers, MA, USA), and rat monoclonal anti-CD34 (ab8158, 1:50, Abcam, Cambridge, UK). The following day, tissue sections were incubated with specific secondary antibodies conjugated to horseradish peroxidase (HRP-linked anti-mouse IgG, #7076, HRP-linked anti-rabbit IgG, #7074 Cell Signaling Technology, Danvers, MA, USA, ab182931 HPR-linked anti-rat IgG) and the SignalStain^®^ DAB Substrate Kit (#8059, Cell Signaling Technology, Danvers, MA, USA). DAB (3,3′-diaminobenzidine) was used for antigen detection. Finally, DAB-positive cells were analyzed and quantified using image analysis software ImageJ (https://fiji.sc, accessed on 3 April 2024) and statistical analysis GraphPad Prism 5.0 Software (https://www.graphpad.com, accessed on 3 April 2024).

### 4.8. Western Blot Analysis of Tumor Masses

Tumor tissue lysates (15 μg of proteins) were separated on a TGX Stain-Free FastCast Acrylamide Kit 10% (#1610183, Bio-Rad, Hercules, CA, USA) by an electrophoretic run, transferred onto a PVDF membrane (Millipore, Burlington, MA, USA), and blocked with 5% milk for 1 h. Then, the membranes were incubated overnight at 4 °C with the following primary antibodies: anti-AVPR2 (rabbit polyclonal PA5-75409, 1:1000, Invitrogen, Waltham, MA, USA), anti-P-AKT (rabbit monoclonal 4058S, 1:1000, Cell Signaling Technology, Danvers, MA, USA), anti-AKT (rabbit monoclonal 9272S, 1:1000, Cell Signaling Technology, Danvers, MA, USA), anti-PKA (mouse monoclonal PA5-21842, 1:500, Invitrogen, Waltham, MA, USA), anti-PCNA (mouse monoclonal #2586S, 1:2000, Cell Signaling Technology, Danvers, MA, USA), anti-CD34 (mouse monoclonal ab8158, 1:50, Abcam, Cambridge, UK), anti-caspase-3 (rabbit polyclonal #9661, 1:1000, Cell Signaling Technology, Danvers, MA, USA), and anti-Vascular Endothelial Growth Factor (VEGF) (rabbit polyclonal 07-1420 Merck Millipore Milan, Milan, Italy). After this time, the incubation with the secondary antibody was carried out (HRP-linked anti-mouse IgG, #7076 Cell Signaling Technology, Danvers, MA, USA or HRP-linked anti-rabbit IgG, G-21234 Invitrogen, Waltham, MA, USA). Bound antibodies were detected using ECL reagents (Millipore, MA, USA) and chemiluminescent images were acquired with a Bio-Rad ChemiDoc Imaging System (Biorad, Hercules, CA, USA). Densitometric analysis was performed using ImageJ Software Java 8. The experiments were performed using all tumor mass extracts. The results of each sample were normalized against the total protein quantity loaded in the gel for the corresponding sample. The normalization process was carried out using a stain-free system.

### 4.9. Statistical Analysis

The Shapiro–Wilk normality test was employed to assess the normality of the data distribution for determining whether parametric or non-parametric statistical tests should be used. The following tests were used to perform the comparative analyses: *t* test, one-way ANOVA, or two-way ANOVA. The Bonferroni test was used to perform multiple comparisons. The Kaplan–Meier method was used to analyze the survival and the significance was analyzed by the log-rank test. All values are expressed as the mean ± standard error (SEM), unless otherwise stated. A value of *p* ≤ 0.05 was considered to indicate a statistically significant difference.

## Figures and Tables

**Table 1:** Biochemical parameters of control- and tolvaptan-treated mice at sacrifice.

	Na^+^ (mEq/L)	ALT (IU/L)	AST (IU/L)	Urea (mg/dL)	Creatinine (mg/dL)	Urea/Creatinine
**Control group**	152.3 ± 0.3	19.3 ± 2.8	97.5 ± 23.0	62.3 ± 4.8	0.09 ± 0.02	8.5 ± 1.4
**Tolvaptan group**	154.8 ± 2.2	20.4 ± 3.1	123.8 ± 15.7	47.0 ± 3.7	0.07 ± 0.01	6.9 ± 1.1

## Data Availability

The data and materials used to support this study are available from the corresponding authors (BenedettaFibbi) upon request.
